# Effect of atorvastatin on the expression of gamma-glutamyl transferase in aortic atherosclerotic plaques of apolipoprotein E–knockout mice

**DOI:** 10.1186/1471-2261-14-145

**Published:** 2014-10-18

**Authors:** Gang Li, Xiao-wei Wu, Wei-hua Lu, Rong Ai, Fang Chen, Zhong-zhi Tang

**Affiliations:** Emergency Department, Wuhan General Hospital of Guangzhou Military Command, Wuhan, 430074 China; Department of Thoracic Surgery, TongJi Hospital, TongJi Medical College, Huazhong University of Science and Technology, Wuhan, China; College of Foreign Language, Huazhong Agriculture University, Wuhan, China; Department of Medicine Laboratory, Wuhan General Hospital of Guangzhou Military Command, Wuhan, China

**Keywords:** Statins, Atherosclerosis, Gamma-glutamyltransferase, Inflammation

## Abstract

**Background:**

Gamma-glutamyl transpeptidase (GGT) is now considered to be one of the risk factors for cardiovascular disease. However, whether statins can alter GGT levels in arterial atheromatous plaque has not yet been studied. Therefore, the aim of this study is to determine whether statins can effectively decrease the expression of GGT in arterial atheromatous plaques.

**Methods:**

We randomly divided 45 apolipoprotein E–knockout (ApoE KO) male mice into three groups: normal diet (ND) group,high-cholesterol diet (HCD) group and high-cholesterol diet and atorvastatin (HCD + Ato) group. We fed high-cholesterol food to the HCD and HCD + Ato group. After eight weeks, atorvastatin 5 mg•kg^−1^•d^−1^ was given to HCD + Ato group mice. The serum GGT-1, intercellular cell adhesion molecule-1 (ICAM-1) and vascular cell-adhesion molecule-1 (VCAM-1) levels were measured at end of 16 weeks by using ELISA methods. The expressions of GGT-1, ICAM-1 and VCAM-1 in aorta were measured by RT-PCR and Western Blot.

**Results:**

The ApoE KO mice with HCD were associated with a marked increase in plasma lipid, inflammatory factors, GGT-1, ICAM-1 and VCAM-1. The expressions of GGT-1, ICAM-1 and VCAM-1 in HCD aortic tissue were increased. At the HCD + Ato group were treated with atorvastatin, the levels of lipid, GGT-1, ICAM-1 and VCAM-1were suppressed. Meanwhile, the expressions of GGT-1, ICAM-1 and VCAM-1 were significantly decreased in the whole aorta plaques.

**Conclusions:**

The effect of statins on the expression of GGT in aorta plaque was firstly observed in animal model. The research shows that statins can significantly decrease the expression of GGT in aortic atherosclerotic plaques.

## Background

In clinical practice statins can be used to decrease low-density lipoprotein (LDL) levels. Cardiovascular events and mortality are decreased significantly by the use of statins [1–3], and statins have already become a standard treatment for coronary heart disease. The mechanisms by which statins may alleviate the symptoms of coronary artery disease may not be limited only to their lipid-lowering effect. Statins also have pleiotropic biological effects that occur before lipid levels decrease. These additional effects may be related to the ability of statins to improve the bioavailability of nitric oxide [4], repair damaged cells [5], promote angiogenesis [6], and function as an anti-inflammatory mediator and antioxidant [7].

However, the occurrence of cardiovascular events in clinical practice cannot be prevented. This is despite being able to control LDL levels, the main risk factor. Research has shown that when the LDL levels are decreased to 2–3 mmol/L after intensive treatment with statins, the risk of cardiovascular events is decreased by 40%–50% [8]. This means that a considerable risk has not been effectively controlled. Residual cardiovascular risk remains that is not managed effectively. This residual cardiovascular risk is both associated with and independent of the presence of high lipid levels. The non-lipid associated risk is considered to mainly be influenced by the levels of uric acid, fibrinogen, C-reactive protein (CRP) and homocysteine [9].

Gamma-glutamyl transpeptidase (GGT) is now considered to be one of the risk factors for cardiovascular disease [10–12]. GGT is the critical enzyme in the gamma-glutamyl cycle. The primary function of GGT is to hydrolyze glutathione, which is a main in vivo antioxidant. Therefore, many studies consider GGT level as anindex of the oxidation state [10, 13, 14]. Serum GGT levels are correlated with multiple risk factors in cardiovascular disease. Furthermore, serum GGT levels can predict the prognosis of cardiovascular diseases and cardiovascular events. A few studies [15] have shown that GGT, located in arterial atheromatous plaques, can promote the oxidation of LDL through a redox reaction and lead to the further development of plaque. Furthermore, whether statins can alter GGT levels in arterial atheromatous plaque has not yet been studied. Therefore, the aim of this study is to determine whether statins can effectively decrease the expression of GGT in arterial atheromatous plaques and discuss the mechanism by which this occurs.

## Methods

### Animals and treatment

This study was approved by the Experimental Animal Ethics Committee of Wuhan General Hospital. Forty-five apolipoprotein E–knockout (ApoE KO) male mice (C57BL/6 J, 6 weeks old, weighing an average of 20.5 g) were bred from breeding pairs obtained from The Jackson Laboratory (Bar Harbor, Maine, USA) by the Animal Center of Peking University. One week after adaptive feeding in a specific pathogen free vivarium, the experimental protocol was initiated. Thirty mice were randomly selected and fed a high cholesterol diet (HCD) that contained 1.25% cholesterin, 10% coconut oil and basic diet. After 8 weeks, the mice in the HCD group were further randomly divided into two groups: 15 mice remained on a high cholesterol diet alone (HCD group) and 15 mice were treated with atorvastatin (HCD and Ato group). The mice in the HCD group were continued on a high cholesterol diet and gavaged with 10 mL/(kg•d) of 0.5% sodium carboxymethyl cellulose (CMC-Na) solution each day. According to the body surface area (BSA) normalization method [16], we converted the drug dose between mice and humans. A dose of 5 mg•kg^−1^•d^−1^ in mice is equivalent to a daily dose of about 25 mg atorvastatin in an adult human subject. The HCD and Ato group were given 5 mg•kg^−1^•d^−1^ atorvastatin dissolved in 0.5% CMC-Na bygavage for 8 weeks. An additional 15 mice were given normal chow for 16 weeks and were referred to as the normal diet group (ND group). All the mice were weighed once a week and the dose of atorvastatin and vehicle adjusted accordingly. After 16 weeks, all of the mice were fasted, anesthetized with 0.5 ~ 1.0 mL of 1% pentobarbital (i.p.) and retinal tissue and blood were collected. The blood was centrifuged at 3000 rpmfor 10 min. The plasma was stored at −80°C. Mice were euthanized and the aortic root of the aortic arch vessels was collected.

### Biochemical measurements

Blood samples were collected from the jugular vein. Blood lipid analysis was performed before and after 8 weeks on the high-cholesterol diet and after 8 weeks of atorvastatin treatment. Plasma totalcholesterol (TC), LDL and total triglyceride (TG) concentrations were measured by an enzymatic method (BioMerieux, Lyon, France) using an automated analyzer (Type 7170A, Hitachi, Japan). Enzyme-linked immunosorbent assay (ELISA) kits were used to measure plasma levels of GGT-1 (Cloud-Clone Crop, USA), intercellular cell adhesion molecule-1 (ICAM-1, RayBiotech, USA), vascular cell-adhesion molecule-1 (VCAM-1, Fitzgerald, USA), interleukin-6 (IL-6, R&D Systems Inc., USA) and high-sensitivity C-reactive protein (hs-CRP, BioVendor R&D Systems Inc., CZ).

### Quantitative detectionof mRNA levels by real time polymerase chain reaction

The whole aorta was milled into a powder in liquid nitrogen and suspended in 1 mLof Trizol. Total RNA was extracted according to the manufacturer’s instructions. cDNA was reverse transcribed with reverse transcriptaseand amplified using the real time polymerase chain reaction (RT-PCR). The glyceraldehyde-3-phosphate dehydrogenase (GAPDH) gene served as the internal reference. The tested gene and GAPDH primers were designed using Primer 5.0 software (Premier Bio, CA) and synthesized using Taq polymerase and reagents from Invitrogen. The primer sequences were: GGT-1: forward, 5′-GGACGTGACCAAGGTGATCT-3′; reverse, 5′-TCGTCCATCTCGTCATTGAA-3′; VCAM-1: forward, 5′-GTGCATCCCCAACATTCTCT-3′; reverse, 5′-TGGTTCTCCAACCTCCAAAG-3′; ICAM-1: forward, 5′-TTGAACAGTGACAGCCCTTG-3′; reverse, 5′-CTCCGTGGGAATGAGACACT-3′; GAPDH: forward, 5′-GCCCTCAATGACCTTTGT-3′; reverse, 5′-AAACTGTGAAGAGGGGCAGA-3′. The reaction conditions were: initial denaturation step for 3 min at 94°C, denature at 94°C for 30s, anneal 30s at 58°C, elongation 30s at 72°C for 30 cycles. A final, elongation step at 72°C for 8 min was included to complete the synthesis of reaction products. Reaction products were separated on a 1% agarose electrophoresis gel and observed under UV light. Samples were measured in duplicate using an Applied Biosystems 7500 Real-Time PCR System. Target gene mRNA levels were calculated and normalized to GAPDH mRNA.

### Western blot determination

Aortic tissue (0.1 g) was homogenized in lysis buffer on an ice-bath until the tissue was completely uniform. The lysate was centrifuged at 9 kr/min for 10 min at 4°C and the supernatant collected. The total protein concentration was measured by the bicinchoninic acid method. Forty microgramsof total protein was added to 2 × SDS buffer solution and denatured by heating at 100°C for 3 min. Proteins were separated by electrophoresis onan 8% SDS-polyacrylamide gel. The total loading quantity of the protein samples was 40 μg per well. Proteins were transferred to nitrocellulose membrane by electroblotting and stained with ponceau to observe the transfer effects and determine the location of the protein molecular weight standards. The membrane was then blocked for 2 h at 37°C with 5% skim milk powder before adding the primary antibody and incubating at 4°C overnight. The primary antibody concentrations were as follows: mouse polyclonal anti-GGT-1 (1:1000 dilution, Santa Cruz, USA), goat polyclonal anti-VCAM-1 (1:1000, R&D Systems), polyclonal anti-ICAM-1 (1:1000 dilution, Santa Cruz, USA). After incubating with the primary antibody the membranes were washed three times in TBST buffer solution. Horseradish Peroxidase (HRP) was added to goat anti-rabbit secondary antibody (1:5000 dilution, Pierce Chemical, USA) with a concentration of 1:3000 to conjugate with HRP at 37°C for 1 h. Membranes were washed an additional three times with TBST buffer solution and developed by X-ray. Alpha Imager HP (Alpha Innotech, USA) gel imaging apparatus was used to acquire images. The results were analyzed with an Alpha Ease Fc (Alpha Imager 3400) image acquisition and analysis system (Alpha Innotech, USA). The target proteinsare expressed as the ratio of the integrated density of the target gene and the GADPH band.

#### Statistics

SPSS version 18.0 (SPSS Inc, Chicago, Illinois) statistical software was used for statistical analysis. Comparisons were performed by Student’s *t* test and ANOVA followed by the Student–Newman–Keuls test. Categorical variables were analyzed using the chi-squared test. Pearson’s correlation analysis was used to examine the correlation between serum GGT-1 and inflammatory factors, hs-CRP and IL-6. A *P* value less than 0.05 was considered significant.

## Results

### Atorvastatin reduced blood lipid, hs-CRP and IL-6

There were no significant differences in plasma lipid, hs-CRP and IL-6 levels among the three groups at baseline. After 8 weeks of a high-cholesterol diet, plasma concentrations of TC, LDL, hs-CRP and IL-6 were significantly increased (P < 0.01). Plasma TG concentrations were not different. After the ApoE KO mice were treated with atorvastatin, the level of TC, LDL, hs-CRP and IL-6 in the HCD and Ato group decreased significantly compared with the HCD group (P < 0.05, Table 1).

**Table 1:** **The influences of atorvastatin on blood lipid, hs-CRP and IL-6**

	Time	ND	HCD	HCD + Ato
**TC(mmo/L)**	0 W	1.51 ± 0.24	1.42 ± 0.34	1.32 ± 0.28
	8 W	1.53 ± 0.17	22.31 ± 1.96*	23.08 ± 1.77*
	16 W	1.49 ± 0.09	26.58 ± 2.08*	20.01 ± 1.16^#^
**TG(mmo/L)**	0 W	1.07 ± 0.51	0.99 ± 1.04	1.11 ± 0.08
	8 W	1.03 ± 0.45	1.24 ± 1.29	1.31 ± 0.78
	16 W	1.05 ± 0.59	1.44 ± 1.28	1.38 ± 0.56
**LDL(mmo/L)**	0 W	0.97 ± 0.14	1.06 ± 0.27	1.01 ± 0.03
	8 W	1.05 ± 0.08	15.33 ± 1.17*	15.88 ± 2.34*
	16 W	0.98 ± 0.33	16.87 ± 1.24*	9.53 ± 1.87^#^
**hs-CRP(mg/ml)**	0 W	2.34 ± 0.13	2.29 ± 0.14	2.33 ± 0.13
	8 W	2.41 ± 0.15	6.58 ± 1.72*	6.71 ± 1.68*
	16 W	2.39 ± 0.15	11.83 ± 2.09*	7.75 ± 1.87^#^
**IL-6(ng/ml)**	0 W	0.17 ± 0.05	0.16 ± 0.04	0.17 ± 0.05
	8 W	0.18 ± 0.05	0.32 ± 0.15*	0.34 ± 0.14*
	16 W	0.17 ± 0.06	0.57 ± 0.21*	0.13 ± 0.08^#^

Apolipoprotein E–knockout mice were treated with ND, HCD, or HCD plus atorvastatin, as described in the Methods section.TC: total cholesterol; TG: total triglyceride; LDL: low-density lipoprotein; HDL: high-density lipoprotein; hs-CRP: high-sensitivity C-reactive protein; IL-6: interleukin-6. ND: normal standard diet; HCD: high-cholesterol diet; Ato: atorvastatin, at 5 mg•kg^−1^•d^−1^. Values are means ± SD, n =15 for each group. *:P < 0.01 compared with ND group; ^#^:P < 0.05 compared with HCD group.

### ELISA and RT-PCR analysis of GGT-1, VCAM-1 and ICAM-1

**Figure 1:**
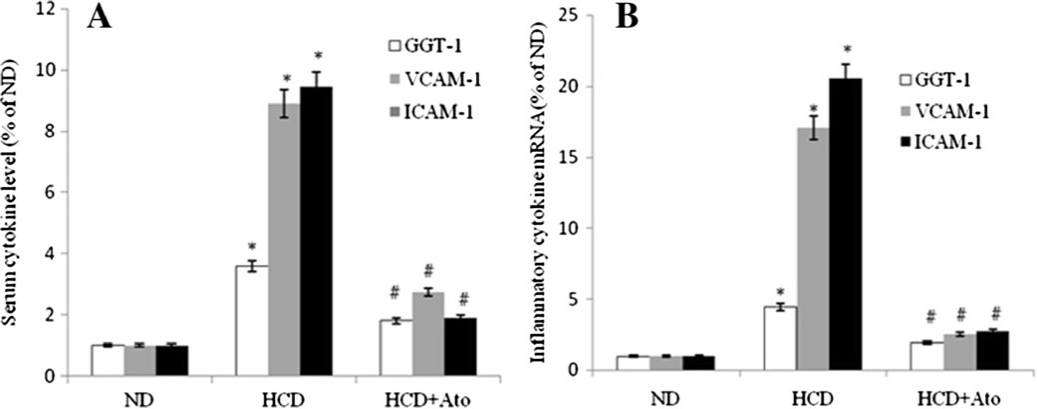
**ELISA and RT-PCR analysis of GGT-1, VCAM-1 and ICAM-1. A**: effect of atorvastatin on GGT-1, VCAM-1and ICAM-1 serum concentrations in ApoE KO mice. Serum GGT-1, VCAM-1and ICAM-1 was measured using an ELISA kit. *P <0.01 as compared with ND group; ^#^P <0.01 as compared with HCD group. **B**: effect of atorvastatin treatment on GGT-1, VCAM-1 and ICAM-1 mRNA expression in arterial atheromatous plaque. The mRNA levels of GGT-1, VCAM-1 and ICAM-1were measured by RT-PCR, as described in the Methods section. *P <0.01 as compared with ND group; ^#^P <0.01 as compared with HCD group.

The plasma concentrations of GGT-1, VCAM-1 and ICAM-1 detected by ELISA in the HCD and Ato group decreased significantly compared with the HCD group (P < 0.001) (Figure 1). Plasma GGT-1 levels had a weak correlation with the level of hs-CRP (r = 0.341, P < 0.001) and IL-6 (r = 0.278, P < 0.001).

Real-time quantitative PCR showed that the expression of GGT-1, VCAM-1, and ICAM-1 increased significantly compared with the ND group (ND group versus HCD group: GGT-1, 1 ± 0.13 versus 4.44 ± 0.84, P < 0.01; VCAM-1, 1 ± 0.18 versus 17.07 ± 1.27, P < 0.01; ICAM-1,1 ± 0.19 versus 20.51 ± 2.07, P < 0.01). However, the expression of GGT-1, VCAM-1, and ICAM-1 decreased significantly in the HCD and Ato group compared with the HCD group (HCD group versus HCD and Ato group, GGT1, 4.44 ± 0.84 versus 1.96 ± 0.39, P < 0.01; VCAM-1, 17.07 ± 1.27 versus 2.51 ± 0.53, P < 0.01; ICAM-1, 20.51 ± 2.07 versus 2.71 ± 0.43, P < 0.01).

### Western blot analysis

**Figure 2:**
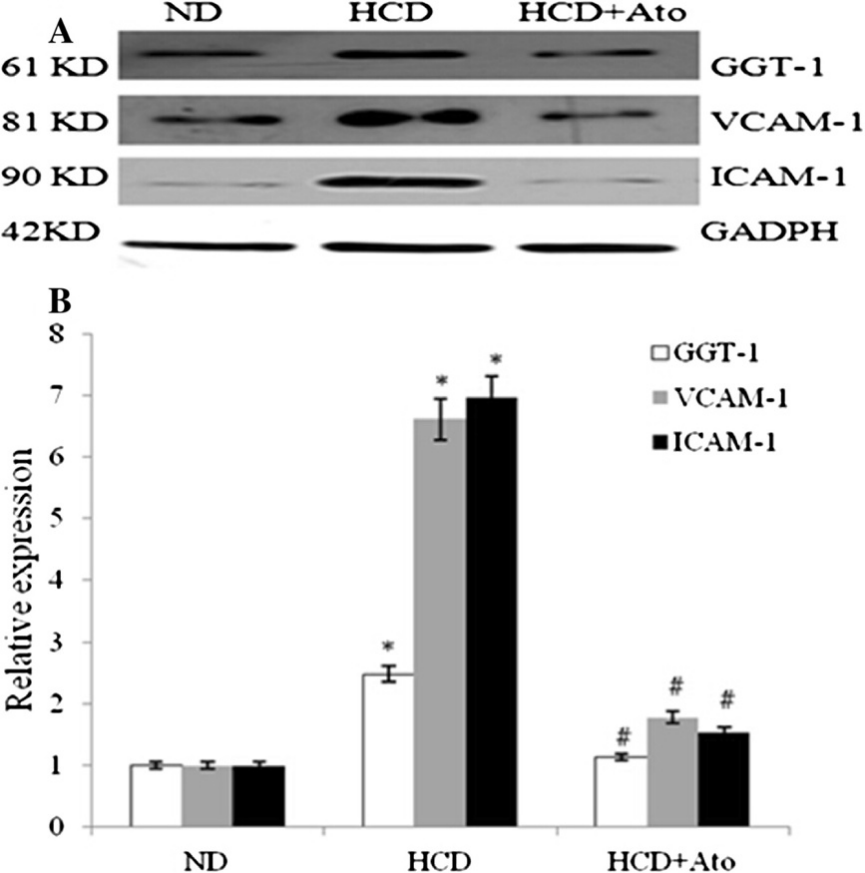
**The protein levels of GGT-1, VCAM-1 and ICAM-1 were measured by Western blots, as described in the** **Methods section** **. A**: densitometric measurements of GGT-1, VCAM-1 and ICAM-1 from Western blots. **B**: one representative result of Western blots of GGT-1, VCAM-1 and ICAM-1 was shown.

The Western blots showed that the expressing levels of GGT-1, VCAM-1 and ICAM-1 inwhole aortic tissuefrom ApoE KO mice decreased sharply compared with the HCD group after 8 weeks of atorvastatin treatment (Figure 2)

## Discussion

Atherosclerosis is a complex, progressive disease resulting from the combined effect of several factors. GGT is the key enzyme to hydrolyze glutathione, amain in vivo antioxidant. In our study, the effect of atorvastatin on the expression of GGT in arterial atheromatous plaques was observed in an animal model. The data collected show that atorvastatin can, simultaneously, decrease the expression of adhesion molecules, VCAM-1 and ICAM-1, in aortic tissue.

Glutathione was hydrolyzed by GGT and resulted in many oxidized substances, such as reactive oxygen species (ROS), oxidizedlow-density lipoprotein (Ox-LDL) and so on [17]. At present, many studies have confirmed that GGT level is an independent risk factor for cardiovascular disease, and it can predict the prognosis of patients with cardiovascular diseases and the occurrence of cardiovascular events [11, 18, 19]. The evidence, that the GGT activity is increased in arterial atheromatous plaques [20], suggests that GGT has the possibility to promote the developmentof arterial atheromatous plaque. Some studies have indicated that the GGT activity in patients with acute coronary syndrome (ACS) was higher than patients with stable coronary artery disease (CAD) [21]. There is also a positive correlation between GGT and the SYNTAX score, which reflects the complexity and severity of the coronary arterylesion [22].

After GGT accumulates in a plaque, the formation of Ox-LDL [17] and inflammation [23] are promoted by an increase in oxidative stress, which contributes to apoptosis, plaque rupture, and the progression and instability of the atherosclerotic plaque. In our study, atorvastatin can significantly decrease the expression of GGT in aortic atherosclerotic plaques. There are several potential mechanisms.

1. Atorvastatin could reduce the interaction of LDL with GGT,and block serum GGT transfer into the atheromatous plaque by decreasing LDL levels in blood. Studies have shown that LDL can be transported with GGT and catalyze activity within plaques [24]. If the level of LDL is reduced, the transport of GGT in the blood into the plaque and the expression of GGT will also be reduced. Our research has found that by decreasing LDL levels in the blood of experimental animals, the expression level of GGT in the plaque decreases significantly at the same time.
2. GGT could be a marker of an increased oxidation state [10, 13, 14]. Atorvastatin could decrease the expression of GGT by reducing inflammatory factors and oxidative stress. CRP and IL-6 are indices that reflect the conditionof atherosclerotic inflammation. Many studies have found that there is a positive correlation between serum GGT levels, CRP and IL-6 [25, 26]. GGT is the main enzyme that could catalyze the metabolism of the thiol antioxidant GSH. Thus, its level might also reflect the degree of inflammation and oxidative stress. When the mice in our experiment were fed with atorvastatin, the levels of GGT, CRP and IL-6 in serum were simultaneously decreased.
3. The expression of VCAM-1 and ICAM-1 were decreased by atorvastatin. Monocytes that adhered to the arterial intima and foam cells decreased. Finally, the accumulation of plaque in the local tissue was decreased. Aldo Paolicchi et al. [15] found mainly CD68^+^ foam cells in the atheromatous plaque,which had GGT catalytic activity. Monocytes in the blood were the main source of foam cells [27]. VCAM and ICAM mediate monocyte attachment to endothelial cells and the arterial wall [28]. This is a critical step in the process of atherosclerosis. Previous studies have also shown that statins can inhibit the expression of VCAM and ICAM in endothelial cells [29, 30]. Therefore, atorvastatin may first inhibit the expression of VCAM and ICAM in endothelial cells that results in decreased monocyte attachment to endothelial cells. Finally, the formation of foam cells and the accumulation of plaque in the local tissue decreased. In our study, the mRNA and protein expression levels of VCAM, ICAM and GGT in thewhole aortic tissue decreased significantly (P < 0.01). This shows that atorvastatin can decrease the content of GGT in the plaque through VCAM and ICAM.

We found that feeding a high fat diet can induce the expression of GGT in atheromatous plaques of ApoE KO mice. There are different mechanisms for atheromatous to inhibit the expression of GGT in plaques. Statins can inhibit the expression of GGT in atheromatous plaque of local tissue, which leads to a decrease in oxidative stress in the plaque. This may promote the stabilization of atherosclerotic plaque and reduce the incidence of cardiovascular events. The results of this study revealed yet another new way for statins to act on plaques.

## Conclusion

In our study, the effect of statins on the expression of GGT in arterial atheromatous plaque was first observed in an animal model. Our findings show that statins can significantly decrease the expression of GGT in aortic atherosclerotic plaques.

## Abbreviations

ApoE KO: Apolipoprotein E–knockout
CAD: Coronary artery disease
ACS: Acute coronary syndrome
ROS: Reactive oxygen species
RT-PCR: Real time polymerase chain reaction
GAPDH: Glyceraldehyde-3-phosphate dehydrogenase
ELISA: Enzyme-linked immunosorbent assay.
